# Longitudinal relationships between daily activities, depressive symptoms, anxiety, and suicidality during the COVID-19 pandemic: a three-wave cross-lagged study

**DOI:** 10.3389/fpubh.2025.1459300

**Published:** 2025-03-11

**Authors:** Juhee Choi, Gaeun Son, Kee-Hong Choi

**Affiliations:** ^1^Department of Psychology, Korea University, Seoul, Republic of Korea; ^2^KU Mind Health Institute, Korea University, Seoul, Republic of Korea; ^3^Mindeep Cognitive Behavioral Therapy Center, Seoul, Republic of Korea

**Keywords:** depressive symptoms, anxiety, suicidality, daily activities, cross-lagged panel model, longitudinal, COVID-19

## Abstract

**Background:**

The COVID-19 pandemic has caused significant unprecedented changes in lives. In particular, core daily activities, such as sleep, diet, physical activity, education, and social activities have significantly changed. Restrictions on daily activities are associated with the deterioration of mental health. However, few studies have comprehensively explored the relationship between daily activities and mental health during the COVID-19 pandemic, and longitudinal studies of these relationships are lacking. Therefore, this study examined the dynamic interaction between core daily activities and mental health during the COVID-19 pandemic.

**Methods:**

An online survey was conducted with 586 adults (age: M = 46.80, SD = 12.515) in three waves: September 2020, December 2020, and April 2021. Depressive symptoms, anxiety, and suicidality were assessed using Mental Health Screening Tool. Daily activities were assessed using Core Life Activities Inventory. Autoregressive and cross-lagged effects were investigated using a cross-lagged panel analysis.

**Results:**

Reduced daily activities contributed to increased depression, anxiety, and suicidality, which, in turn, resulted in further decreases in daily activities in subsequent waves. These autoregressive and reciprocal effects persisted for 7 months during the pandemic. Among core daily activities, sleep quality, physical activity, and social activities had reciprocal relationships with depression. Moreover, only social activities showed a reciprocal relationship with anxiety and suicidality.

**Conclusion:**

These findings highlight the complex relationship between core daily activities and mental health and provide valuable insights for targeted therapeutic strategies. Implementing timely and effective interventions to maintain and enhance key activities, particularly social engagement, is crucial for alleviating negative mood symptoms. Clinical support and promotion of these essential daily activities are necessary to improve mental health outcomes.

## Introduction

The novel coronavirus (COVID-19) was first detected in Wuhan Province, China, in December 2019 (1). The outbreak of the COVID-19 pandemic led to unprecedented challenges that affected various aspects of daily life globally (2). To curb the spread of the virus, governments enacted stringent regulations, including lockdowns, for individuals exhibiting symptoms or those in contact with them. Public health strategies, such as social distancing, home confinement, and lifestyle changes significantly affected mental health (3). Quarantine and social distancing measures constrained daily activities, deteriorating mental wellbeing (4–6).

Five key daily activities—sleep, diet, exercise, education, and social connections—are particularly important in mental and physical health (7–10). Extended home confinement caused increased issues with sleep, including poor sleep quality and symptoms of sleep disorders (11, 12). Moreover, changes in dietary habits emerged, marked by increased snacking, late-night eating, weight gain, and an increase in home-cooked meals (8, 13). Physical activity decreased, whereas sedentary behavior increased (14, 15). Furthermore, the pandemic disrupted global education systems (16) and severely restricted social gatherings.

Deficiencies in daily activities increased depression and anxiety, and depression and anxiety reduced daily activities. One study found that these reciprocal autoregressive effects persisted throughout a seven-month period of the pandemic (17). A meta-analysis of 0.9 million people across 32 countries found that disrupted daily routines were strongly associated with depressive symptoms and anxiety (18). For instance, sleep disturbance, a common feature of depression and anxiety (19, 20), exhibited a bidirectional relationship with disrupted daily routines (21). Poor sleep and diet quality were correlated with negative moods (22). Sleep disturbances, including nightmares and insomnia showed a significant link between suicidal ideation, suicide attempts, and actual suicides, as found in longitudinal studies (23, 24). Additionally, there was much evidence of associations between poor dietary quality and depression, anxiety, and suicidal ideation (25). Physical inactivity during the pandemic was linked to increased depression scores (26), and reduced social contact and mobility were associated with higher levels of depression and anxiety (27). Reduced physical activity combined with increased sedentary behavior substantially raised the risk of suicidal ideation and planning among adolescents of both genders and correlated with a greater frequency of suicide attempts in males, compared to those maintaining sufficient physical activity and lower levels of sedentary behavior (28, 29). Solitude and the experience of loneliness were significantly associated with suicidal ideation and attempts, as found in research (30).

Disruption of daily activities was found to be correlated with increased symptoms of depression and anxiety and increased suicidal thoughts and behaviors (31, 32). Although previous studies have highlighted the link between lifestyle factors and mental health, they mainly focused on a single factor, and few have examined the comprehensive relationship between activities in daily life and mental health outcomes. Moreover, most studies have relied on cross-sectional designs that lack the longitudinal and dynamic perspectives necessary to understand the evolving relationships during a global health crisis.

Therefore, this study examined the autoregressive, temporal, and reciprocal relationships between daily activities and mental health during the COVID-19 pandemic using a three-wave cross-lagged analysis. In contrast to cross-sectional designs, cross-lagged analyses evaluate multiple time points, rendering them particularly suitable for determining the directionality of relationships between lifestyle factors and mental health states. By integrating autoregressive and cross-lagged pathways, these analyses provide insights into the stability and bidirectional effects of lifestyle factors (33). Moreover, this study aimed to identify the impact of demographic factors, such as age, gender, income, and educational status, on these relationships. The hypotheses are that lower levels of daily activities are associated with higher levels of depression, anxiety, and suicidality in subsequent periods. Conversely, higher levels of depression, anxiety, and suicidality are associated with lower levels of daily activities across three waves.

## Methods

### Participants and design

We conducted an online questionnaire survey on daily activities, depression, anxiety, and suicide risk among adults in South Korea. We recruited representative samples of participants in three waves: September 1–8, 2020 (Wave 1), December 16–23, 2020 (Wave 2), and April 19–28, 2021 (Wave 3). Participants aged 18–68 years were recruited through an online research company using a stratified sampling method based on age, gender, and geographic area. No other exclusion criteria were applied. A total of 817 individuals responded to the survey (Wave 1: 817, Wave 2: 684, and Wave 3: 586). The data of participants who provided complete responses in all three waves (*n* = 586) were analyzed. This study was approved by the Institutional Review Board of Korea University (KUIRB-2021-0013-02). All participants voluntarily provided written online informed consent prior to participating in the study. Participants were compensated with 1,500 KRW (roughly 1.12 USD) for completing each survey.

### Measures

#### Anxiety

Anxiety was measured using the 11-item Mental Health Screening Tool for Anxiety Disorders (MHS-A) (34), which evaluates respondents’ anxiety over the preceding 2 weeks. Responses are rated on a 5-point Likert scale (0 = never; 4 = most of the time). The Cronbach’s alpha coefficients for the MHS-A in Waves 1–3 were 0.962, 0.969, and 0.964, respectively, indicating good internal consistency.

#### Depressive symptoms

Depressive symptoms were measured using the 12-item Mental Health Screening Tool for Depressive Disorders (MHS-D) (35), which evaluates respondents’ depression symptoms over the preceding two weeks. Responses are rated on a 5-point Likert scale (0 = not at all; 4 = strongly agree). The Cronbach’s alpha coefficients for the MHS-D for Waves 1–3 were 0.947, 0.947, and 0.946, respectively, indicating good internal consistency.

#### Suicidality

Suicidality was measured using the four-item Mental Health Screening Tool for Suicide Risk (MHS-S) (36), which evaluates respondents’ willingness to live, suicidal ideation, and consideration or plans to commit suicide over the preceding two weeks as well as previous suicide attempts. Responses are rated on a 5-point Likert scale (0 = never; 4 = always). The Cronbach’s alpha coefficients for the MHS-S in Waves 1–3 were 0.909, 0.917, and 0.917, respectively, indicating good internal consistency.

#### Daily activities

Daily activities were measured using the five-item Core Life Activities Inventory (CORE) (7), which evaluates respondents’ sleep quality, diet, physical activity, education, and social activities over the preceding week. Responses are rated on a 5-point Likert scale (1 = not at all; 5 = strongly agree). The Cronbach’s alpha coefficients for CORE in Waves 1–3 were 0.767, 0.776, and 0.815, respectively, indicating good internal consistency.

### Statistical analysis

Tables 1 and 2 present the descriptive statistics, and Table 3 presents the correlations among the research variables. Pearson’s correlation analysis was conducted using SPSS 25.0 to investigate cross-sectional and longitudinal associations among the variables.

**Table 1:** Sample demographics.

	Total sample (*N* = 586)
	M (SD)
Age	46.80 (12.515)
	*N* (%)
Gender	
Male	295 (50.3%)
Female	291 (49.7%)
Marital status	
Single	184 (31.4%)
Married	360 (61.43%)
Divorced	22 (3.75%)
Widowed	16 (2.73%)
Unreported	4 (0.68%)
Educational level	
Elementary schools	3 (0.51%)
middle schools	3 (0.51%)
high schools	104 (17.75%)
undergraduate	406 (69.28%)
More than graduate	70 (11.95%)

**Table 2:** Descriptive statistics.

Measure	Min	Max	M(SD)
MHS: D			
W1	0	50.66	9.86 (10.6)
W2	0	48.10	9.31 (10.11)
W3	0	51.64	8.5 (9.82)
MHS: A			
W1	0	42.04	9.35 (9.93)
W2	0	45.96	8.65 (10.06)
W3	0	39.76	8.35 (9.49)
MHS: S			
W1	0	16	1.16 (2.64)
W2	0	16	1.09 (2.62)
W3	0	16	1.12 (2.62)
CORE			
W1	5	25	14.94 (3.64)
W2	5	25	14.79 (3.51)
W3	5	25	15.39 (3.72)

**Table 3:** Bivariate correlations.

	1	2	3	4	5	6	7	8	9	10	11	12	13	14	15	16	17	18	19	20	21	22	23	24
1	1																							
2	0.706^**^	1																						
3	0.653^**^	0.717^**^	1																					
4	0.876^**^	0.685^**^	0.636^**^	1																				
5	0.695^**^	0.882^**^	0.678^**^	0.741^**^	1																			
6	0.632^**^	0.696^**^	0.885^**^	0.700^**^	0.729^**^	1																		
7	0.696^**^	0.571^**^	0.546^**^	0.593^**^	0.510^**^	0.462^**^	1																	
8	0.598^**^	0.735^**^	0.558^**^	0.518^**^	0.657^**^	0.477^**^	0.700^**^	1																
9	0.546^**^	0.587^**^	0.715^**^	0.478^**^	0.525^**^	0.613^**^	0.667^**^	0.692^**^	1															
10	−0.267^**^	−0.265^**^	−0.295^**^	−0.329^**^	−0.294^**^	−0.319^**^	−0.109^**^	−0.125^**^	−0.149^**^	1														
11	−0.264^**^	−0.247^**^	−0.266^**^	−0.301^**^	−0.255^**^	−0.255^**^	−0.106^*^	−0.108^**^	−0.116^**^	0.560^**^	1													
12	−0.150^**^	−0.140^**^	−0.134^**^	−0.183^**^	−0.175^**^	−0.162^**^	0.009	−0.017	−0.024	0.442^**^	0.491^**^	1												
13	−0.169^**^	−0.198^**^	−0.230^**^	−0.160^**^	−0.176^**^	−0.201^**^	−0.114^**^	−0.118^**^	−0.107^**^	0.316^**^	0.352^**^	0.374^**^	1											
14	−0.072	−0.076	−0.131^**^	−0.068	−0.066	−0.129^**^	0.050	0.011	−0.006	0.334^**^	0.278^**^	0.439^**^	0.414^**^	1										
15	−0.294^**^	−0.326^**^	−0.330^**^	−0.316^**^	−0.354^**^	−0.344^**^	−0.161^**^	−0.101^*^	−0.161^**^	0.559^**^	0.360^**^	0.251^**^	0.191^**^	0.197^**^	1									
16	−0.298^**^	−0.361^**^	−0.339^**^	−0.316^**^	−0.371^**^	−0.330^**^	−0.178^**^	−0.183^**^	−0.194^**^	0.406^**^	0.560^**^	0.356^**^	0.261^**^	0.182^**^	0.584^**^	1								
17	−0.152^**^	−0.189^**^	−0.212^**^	−0.193^**^	−0.209^**^	−0.215^**^	0.002	0.035	−0.009	0.244^**^	0.296^**^	0.450^**^	0.196^**^	0.280^**^	0.395^**^	0.492^**^	1							
18	−0.198^**^	−0.238^**^	−0.276^**^	−0.188^**^	−0.190^**^	−0.240^**^	−0.178^**^	−0.101^*^	−0.176^**^	0.250^**^	0.233^**^	0.234^**^	0.401^**^	0.227^**^	0.344^**^	0.403^**^	0.384^**^	1						
19	−0.113^**^	−0.114^**^	−0.138^**^	−0.115^**^	−0.111^**^	−0.165^**^	0.011	0.041	−0.003	0.234^**^	0.163^**^	0.257^**^	0.184^**^	0.458^**^	0.364^**^	0.300^**^	0.474^**^	0.365^**^	1					
20	−0.269^**^	−0.358^**^	−0.373^**^	−0.315^**^	−0.343^**^	−0.405^**^	−0.158^**^	−0.164^**^	−0.140^**^	0.523^**^	0.367^**^	0.307^**^	0.212^**^	0.244^**^	0.541^**^	0.408^**^	0.296^**^	0.268^**^	0.216^**^	1				
21	−0.294^**^	−0.323^**^	−0.390^**^	−0.304^**^	−0.299^**^	−0.372^**^	−0.196^**^	−0.171^**^	−0.170^**^	0.346^**^	0.512^**^	0.312^**^	0.255^**^	0.216^**^	0.397^**^	0.560^**^	0.348^**^	0.274^**^	0.192^**^	0.642^**^	1			
22	−0.164^**^	−0.227^**^	−0.230^**^	−0.171^**^	−0.183^**^	−0.221^**^	−0.082^*^	−0.083^*^	−0.065	0.223^**^	0.340^**^	0.437^**^	0.254^**^	0.267^**^	0.271^**^	0.396^**^	0.485^**^	0.243^**^	0.237^**^	0.456^**^	0.593^**^	1		
23	−0.223^**^	−0.303^**^	−0.320^**^	−0.203^**^	−0.244^**^	−0.284^**^	−0.189^**^	−0.202^**^	−0.202^**^	0.199^**^	0.209^**^	0.203^**^	0.391^**^	0.285^**^	0.241^**^	0.289^**^	0.222^**^	0.436^**^	0.174^**^	0.385^**^	0.452^**^	0.478^**^	1	
24	−0.122^**^	−0.148^**^	−0.193^**^	−0.095^*^	−0.100^*^	−0.169^**^	−0.050	−0.058	−0.008	0.264^**^	0.254^**^	0.255^**^	0.260^**^	0.432^**^	0.304^**^	0.238^**^	0.270^**^	0.216^**^	0.481^**^	0.398^**^	0.428^**^	0.447^**^	0.433^**^	1

1. Depression T1, 2. Depression T2, 3. Depression T3, 4. Anxiety T1, 5. Anxiety T2, 6. Anxiety T3, 7. Suicidality T1, 8. Suicidality T2, 9. Suicidality T3, 10. Sleep Quality T1, 11. Diet Quality T1, 12. Physical Activity T1, 13. Social Activity T1, 14. Education T1, 15. Sleep quality T2, 16. Diet quality T2, 17. Physical Activity T2, 18. Social Activity T2, 19. Education T2, 20. Sleep quality T3, 21. Diet quality T3, 22. Physical Activity T3, 23. Social Activity, 24. Education T3. *p < 0.05, **p < 0.01, ***p < 0.001.

To explore the reciprocal and cross-sectional relationships between variables, we used structural equation modeling (SEM) for cross-lagged panel models (CLPMs) in a three-stage process. First, we validated the measurement model. We established confirmatory factor analysis models for the variables (depression, anxiety, suicidality, and daily activities) for each wave. We evaluated model fit using the comparative fit index (CFI), standardized root mean squared residual (SRMR), and root mean square error of approximation (RMSEA). CFI ≥ 0.90, SRMR and RMSEA ≤0.08 indicate acceptable model fit (37, 38).

Next, we evaluated measurement invariance across time for all main variables. Variations in response patterns to survey questions across time points might reflect distinct latent constructs or measurement errors. Therefore, measurement invariance is necessary for valid comparisons over time (39). Alignment of measurement invariance is recommended, as it allows for latent mean differences and comparisons across groups in large longitudinal samples (40).

Finally, we constructed autoregressive CLPMs using SEM in Mplus Version 8.3 (41). CLPMs facilitate the examination of autoregressive, reciprocal, and causal relationships over time while controlling for both autoregressive and cross-sectional effects (42).

## Results

### Preliminary analyses

Little’s Missing Completely at Random (MCAR) test [*χ*^2^(12) = 19.974, *p* = 0.068] revealed that characteristics of missing data were random (43). The independent *t*-test analysis found no significant differences in gender, age, or income between the dropout and final samples. This indicated that missing data were not dependent on specific variables, supporting the listwise approach (44). As missing data accounted for 28.2% (*n* = 231) of the total data, using methods to impute missing values could lead to inaccurate results. The listwise method can be suitable for this data, as it minimizes the risk of biased results. The participants with missing data were excluded from the hypothetical analysis.

### Descriptive statistics and bivariate correlations

The participants’ demographic information is presented in Table 1. Of the 586 participants, 295 (50.3%) were men and 291 (49.7%) were women. Their age ranged from 20 to 68 years, with a mean age of 46.80 (SD = 12.515) years.

Depressive symptoms, anxiety, suicidality, and daily activities were significantly correlated throughout the study period. The descriptive statistics and bivariate correlations are presented in Tables 2 and 3, respectively.

The mental health prevalence rates for each period were measured. The cutoff values for depression, anxiety, and suicidality levels are shown in Supplementary Table S1. Of the participants, 27.8, 24.4, and 23.4% were classified as having moderate-to-severe depressive symptoms in Waves 1–3, respectively; 26.3, 24.2, and 21.7% were classified as having moderate-to-severe anxiety in Waves 1–3, respectively; and 19.1, 16.2, and 18.9% were classified as having moderate-to-severe suicidality in Waves 1–3, respectively.

### Measurement invariance

In longitudinal studies, time invariance is a critical assumption for panel models, ensuring an accurate interpretation of temporal changes. This study assessed the measurement invariance of the latent constructs (depression, anxiety, suicidality, and daily activities) using alignment of measurement invariance. This approach was used to verify the absence of latent mean differences across the three waves before testing the main hypotheses. Each latent variable was independently verified using alignment of measurement invariance. The configural model fit indices indicated satisfactory results for each construct; comprehensive details are provided in Supplementary Table S2. These findings demonstrated longitudinal measurement invariance across all assessments, indicating a consistent measurement of constructs over time.

### Cross-lagged results

#### Cross-lagged panel analysis

Cross-lagged regression models were used to analyze the causal associations among depressive symptoms, anxiety, suicidality, and daily activities, controlling for demographic variables. First, the paths represented the temporal stability of each variable. Second, the paths represented the cross-lagged effects between variables. Finally, the specific daily activities model represented the paths of the demographic variables.

#### General daily activities and mental health

**Figure 1:**
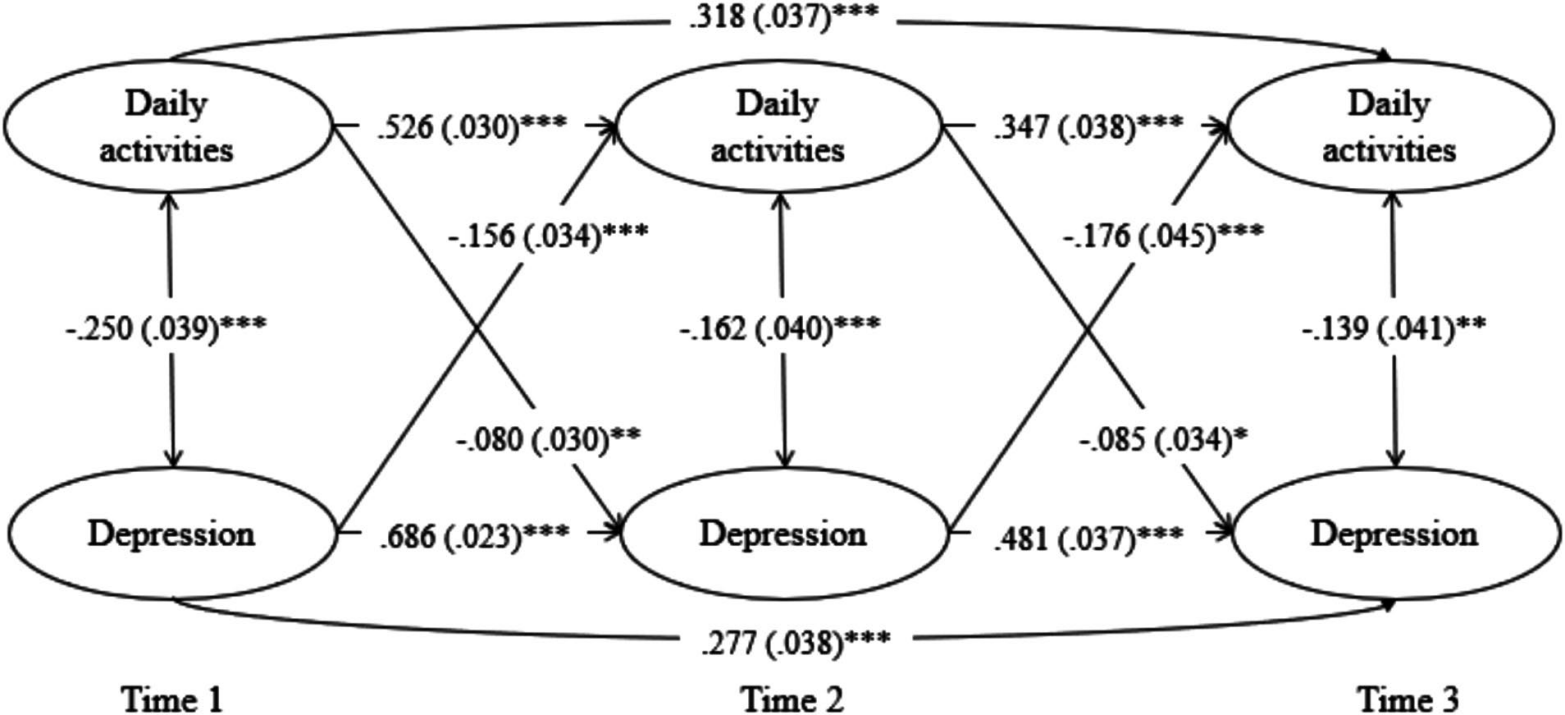
Cross-lagged effects between daily activities and depression.

The CLPM for depressive symptoms and general daily activities exhibited a good fit (Table 4) and significant autoregressive and cross-lagged paths. This implies reciprocal effects, with depressive symptoms in Wave 1 predicting high depressive symptoms (*β* = 0.686, *p* < 0.001) and low daily activities in Wave 2 (*β* = −0.156, *p* < 0.001). Daily activity level in Wave 1 predicted depressive symptoms (*β* = −0.080, *p* = 0.008) and level of daily activities in Wave 2 (*β* = 0.526, *p* < 0.001). Subsequently, depressive symptoms in Wave 2 predicted depressive symptoms (*β* = 0.481, *p* < 0.001) and level of daily activities in Wave 3 (*β* = −0.176, *p* < 0.001). Moreover, the level of daily activities in Wave 2 predicted depressive symptoms (*β* = −0.085, *p* = 0.012) and level of daily activities in Wave 3 (*β* = 0.347, *p* < 0.001). Depressive symptoms in Wave 1 persisted in Wave 3 (*β* = 0.227, *p* < 0.001), and daily activities in Wave 1 influenced those in Wave 3 (*β* = 0.318, *p* < 0.001). However, daily activities and depressive symptoms did not cross-influence each other between Waves 1 and 3 (Figure 1; Table 5).

**Table 4:** Fit indices for the CLPM model of mental health outcomes and general daily activities.

Model specification	*df*	*χ* ^2^	CFI	SRMR	RMSEA
Depressive symptoms Model	24	68.976	0.971	0.069	0.057
Anxiety symptoms Model	24	67.697	0.973	0.066	0.056
Suicidality Model	24	67.008	0.970	0.063	0.055

**Table 5:** Standardized Estimates for the CLPM Model of depression and general daily activities.

Dependent variable	Independent variable	Estimate	S.E.	Est./S.E.	*p*
Depression at T3	Depression at T2	0.481	0.037	12.986	<0.001
	Daily activities at T2	−0.085	0.034	−2.512	0.012
	Depression at T1	0.277	0.038	7.311	<0.001
	Daily activities at T1	−0.050	0.033	−1.507	0.132
Daily activities level at T3	Depression at T2	−0.176	0.045	−3.945	<0.001
	Daily activities at T2	0.347	0.038	9.230	<0.001
	Depression at T1	0.024	0.044	0.540	0.589
	Daily activities at T1	0.318	0.037	8.613	<0.001
Depression at T2	Depression at T1	0.686	0.023	30.366	<0.001
	Daily activities at T1	−0.080	0.03	−2.657	0.008
Daily activities level at T2	Depression at T1	−0.156	0.034	−4.541	<0.001
	Daily activities at T1	0.526	0.03	17.549	<0.001
Daily activities level at T1 WITH	Depression at T1	−0.250	0.039	−6.447	<0.001
Daily activities level at T2 WITH	Depression at T2	−0.162	0.040	−4.029	<0.001
Daily activities level at T3 WITH	Depression at T3	−0.139	0.041	−3.425	0.001

**Figure 2:**
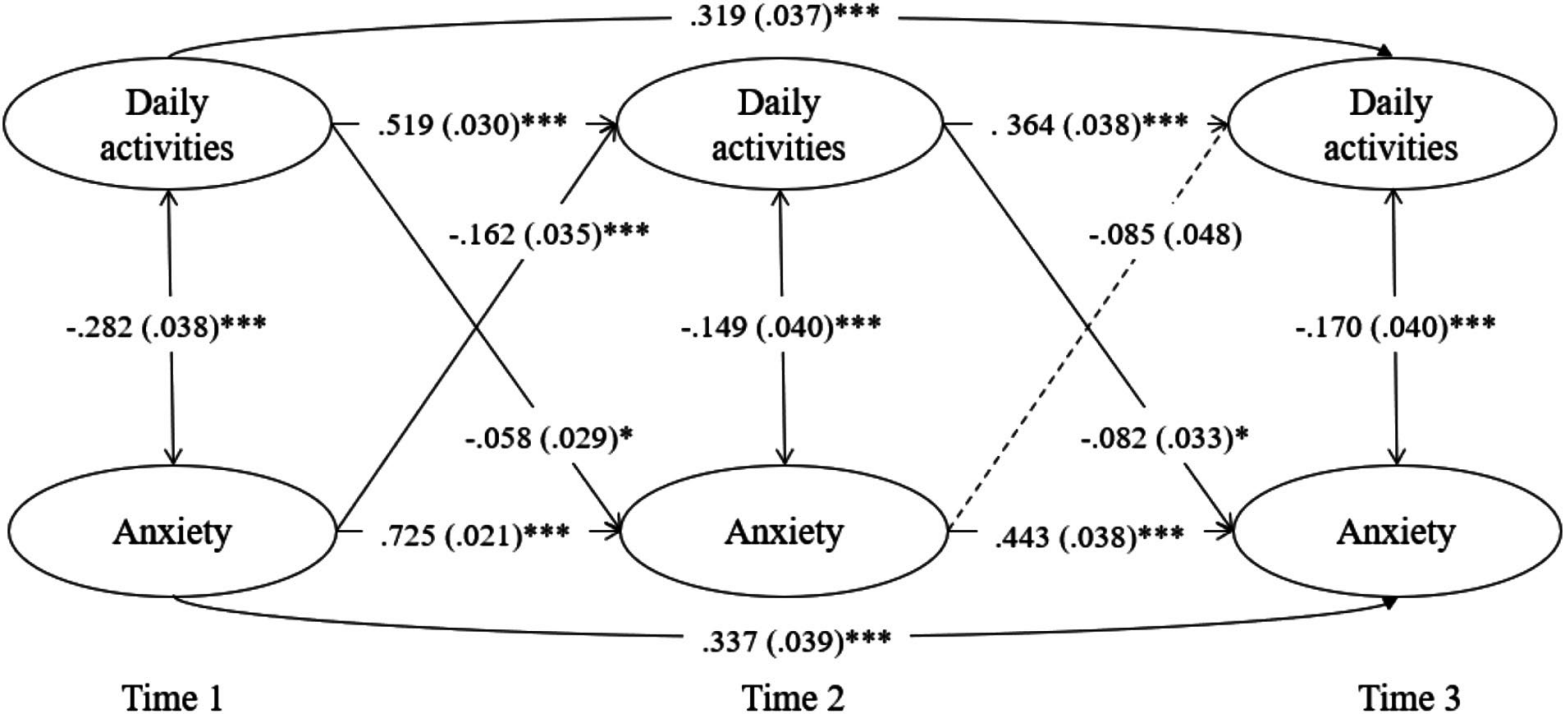
Cross-lagged effects between daily activities and anxiety.

The CLPM for anxiety exhibited a good fit (Table 4) and stable patterns with significant autoregressive and cross-lagged paths. Significant reciprocal effects were observed, with anxiety in Wave 1 predicting high anxiety (*β* = 0.725, *p* < 0.001) and low daily activities in Wave 2 (*β* = −0.162, *p* < 0.001). Daily activity level in Wave 1 predicted anxiety (*β* = −0.058, *p* = 0.044) and level of daily activities in Wave 2 (*β* = 0.519, *p* < 0.001). Subsequently, anxiety in Wave 2 predicted anxiety in Wave 3 (*β* = 0.443, *p* < 0.001) but did not significantly predict level of daily activities in Wave 3 (*β* = −0.085, *p* = 0.075). Moreover, the level of daily activities in Wave 2 predicted anxiety 3 (*β* = −0.082, *p* = 0.013) and level of daily activities in Wave 3 (*β* = 0.364, *p* < 0.001). Anxiety in Wave 1 persisted in Wave 3 (*β* = 0.337, *p* < 0.001), and daily activities in Wave 1 influenced those in Wave 3 (*β* = 0.319, *p* < 0.001). However, daily activities and anxiety did not cross-influence each other between Waves 1 and 3 (Figure 2; Table 6).

**Table 6:** Standardized estimates for the CLPM model of anxiety and general daily activities.

Dependent variable	Independent variable	Estimate	S.E.	Est./S.E.	*p*
Anxiety at T3	Anxiety at T2	0.443	0.038	11.604	<0.001
	Daily activities at T2	−0.082	0.033	−2.493	0.013
	Anxiety at T1	0.337	0.039	8.727	<0.001
	Daily activities at T1	−0.034	0.032	−1.052	0.293
Daily activities level at T3	Anxiety at T2	−0.085	0.048	−1.778	0.075
	Daily activities at T2	0.364	0.038	9.623	<0.001
	Anxiety at T1	−0.019	0.047	−0.391	0.696
	Daily activities at T1	0.319	0.037	8.517	<0.001
Anxiety at T2	Anxiety at T1	0.725	0.021	34.874	<0.001
	Daily activities at T1	−0.058	0.029	−2.014	0.044
Daily activities level at T2	Anxiety at T1	−0.162	0.035	−4.685	<0.001
	Daily activities at T1	0.519	0.03	17.054	<0.001
Daily activities level at T1 WITH	Anxiety at T1	−0.282	0.038	−7.413	<0.001
Daily activities level at T2 WITH	Anxiety at T2	−0.149	0.040	−3.697	<0.001
Daily activities level at T3 WITH	Anxiety at T3	−0.170	0.040	−4.240	<0.001

**Figure 3:**
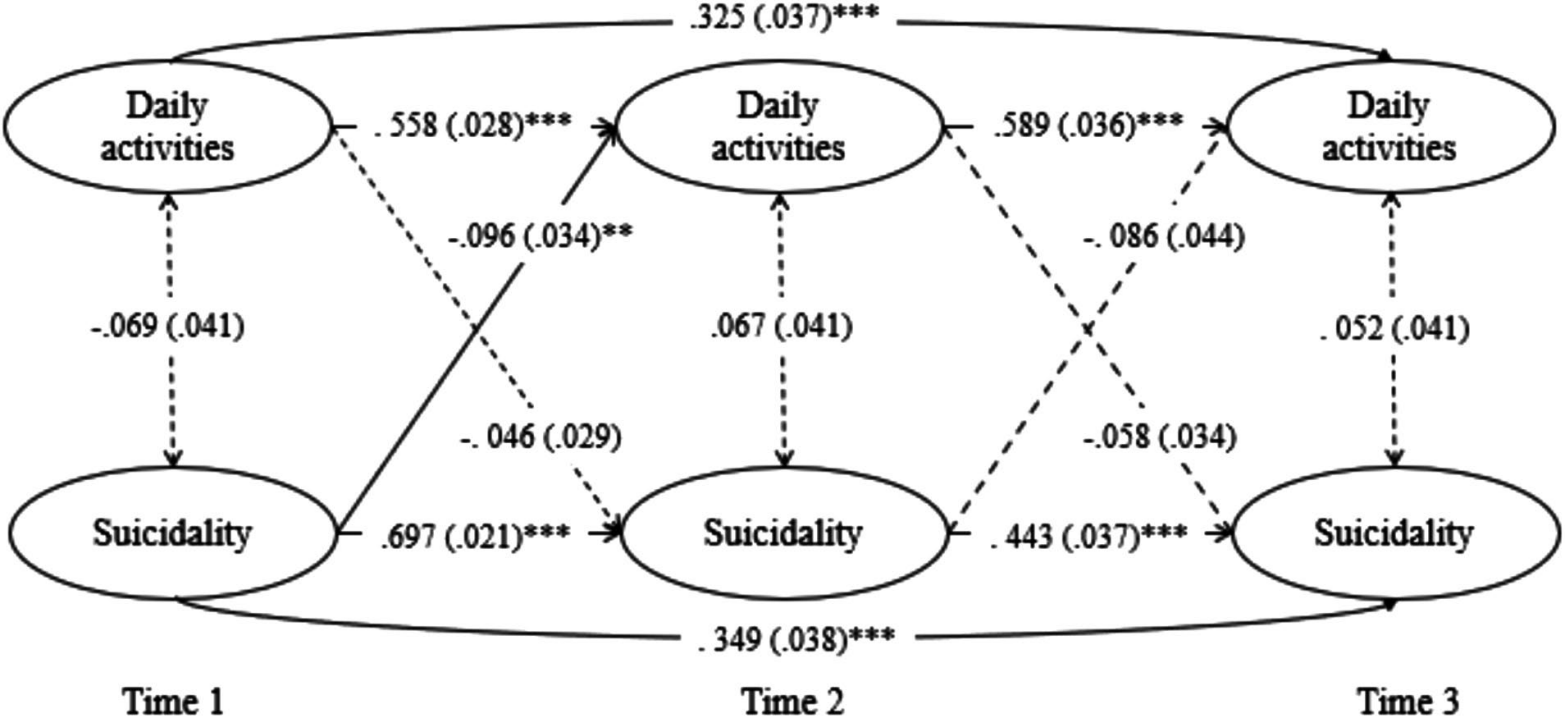
Cross-lagged effects between daily activities and suicidality.

The CLPM for suicidality exhibited a good fit (Table 4) and reciprocal patterns with significant autoregressive and partial cross-lagged paths. Significant stable effects were observed, with suicidality in Wave 1 predicting high suicidality (*β* = 0.697, *p* < 0.001) and low level of daily activities in Wave 2 (*β* = −0.096, *p* = 0.005). Daily activity level in Wave 1 predicted daily activity level in Wave 2 (*β* = 0.558, *p* < 0.001) but did not significantly predict suicidality in Wave 2 (*β* = −0.046, *p* = 0.117). Subsequently, suicidality in Wave 2 predicted suicidality in Wave 3 (*β* = 0.443, *p* < 0.001) but did not significantly influence daily activities in Wave 3 (*β* = −0.086, *p* = 0.051). Moreover, daily activity level in Wave 2 predicted daily activity level in Wave 3 (*β* = 0.383, *p* < 0.001) but did not significantly predict suicidality in Wave 3 (*β* = −0.058, *p* = 0.088). Suicidality in Wave 1 persisted in Wave 3 (*β* = 0.349, *p* < 0.001), and daily activities in Wave 1 influenced those in Wave 3 (*β* = 0.325, *p* < 0.001). However, daily activities and suicidality did not cross-influence each other between Waves 1 and 3 (Figure 3; Table 7).

**Table 7:** Standardized estimates for the CLPM Model of suicidality and general daily activities.

Dependent Variable	Independent Variable	Estimate	S.E.	Est./S.E.	*p*
Suicidality at T3	Suicidality at T2	0.443	0.037	11.84	<0.001
	Daily activities at T2	−0.058	0.034	−1.705	0.088
	Suicidality at T1	0.349	0.038	9.100	<0.001
	Daily activities at T1	−0.008	0.034	−0.251	0.802
Daily activities level at T3	Suicidality at T2	−0.086	0.044	−1.950	0.051
	Daily activities at T2	0.383	0.037	10.419	<0.001
	Suicidality at T1	−0.040	0.044	−0.913	0.361
	Daily activities at T1	0.325	0.037	8.774	<0.001
Suicidality at T2	Suicidality at T1	0.697	0.021	32.844	<0.001
	Daily activities at T1	−0.046	0.029	−1.569	0.117
Daily activities level at T2	Suicidality at T1	−0.096	0.034	−2.840	0.005
	Daily activities at T1	0.558	0.028	19.742	<0.001
Daily activities level at T1 WITH	Suicidality at T1	−0.069	0.041	−1.679	0.093
Daily activities level at T2 WITH	Suicidality at T2	0.067	0.041	1.631	0.103
Daily activities level at T3 WITH	Suicidality at T3	0.052	0.041	1.254	0.210

#### Specific daily activities and mental health

We analyzed the impact of five daily activities on negative mental health outcomes. The model of mental health and daily activities exhibited a good fit (Supplementary Table S3). Individual daily activities exhibited autoregressive paths between the study waves (Supplementary Tables S4–S6). However, few cross-lagged paths with mental health were identified.

Significant autoregressive and cross-lagged paths were identified between depression and sleep, physical activities, and social activities. Diet did not affect depression, and educational activities partially affected subsequent depressive symptoms. This indicated that sleep quality impacted subsequent sleep quality, and depressive symptoms impacted subsequent depressive symptoms. Furthermore, significant reciprocal negative effects were observed, with sleep quality predicting subsequent depression levels, and the severity of depression affecting subsequent sleep quality. Physical activity and depressive symptoms showed significant autoregressive pathways. Moreover, physical activity was partially and reciprocally negatively correlated with depression. In other words, physical activity influenced subsequent depression levels, and the severity of depression affected subsequent physical activity. The relationship between social activities and depressive symptoms showed significant autoregressive paths between each social activities and depressive symptoms, and social activity was negatively associated with depression. That is, social activities influenced subsequent depression levels, and the severity of depression affected subsequent social activities (Supplementary Table S4).

Only social activities exhibited a reciprocal effect on anxiety. This indicates partially reciprocal negative effects, with social activities predicting subsequent anxiety levels, and severity of anxiety affecting subsequent social activities. That is, a higher level of social activities predicted lower levels of anxiety, and more severe anxiety resulted in fewer social activities (Supplementary Table S5).

In the models of suicidality and daily activities, only social activities showed reciprocal effects on suicidality. Higher social activities predicted a lower level of suicidality in the future. The other activities did not show cross-lagged paths to suicidality (Supplementary Table S6).

In addition to demographic variables, lower income and educational status were associated with higher levels of depression, lower income was associated with higher anxiety, and none of the demographic variables were associated with suicidality. Regarding the relationship between demographic variables and specific daily activities, men engaged in more physical activities, whereas women engaged in more social activities. Older participants engaged in more physical activities compared with younger participants (Supplementary Tables S4–S6).

## Discussion

This study examined the longitudinal reciprocal relationships between daily activities and mental health, including depressive symptoms, anxiety, and suicidality. Our findings revealed the stable and longitudinal effects of daily activities, depressive symptoms, anxiety, and suicidality, controlling for age, gender, income, and educational status. Over a seven-month period, general daily activities negatively affected the severity of depressive symptoms, anxiety, and suicidality. Moreover, the severity of mental health issues negatively affected daily activities. Reduced engagement in daily activities was associated with increased severity of depressive symptoms, anxiety, and suicidality, which, in turn, decreased activity levels. In addition, poor mental health was linked to the subsequent worsening of mental health and further decline in critical daily activities. Previous cross-sectional studies have identified a significant negative correlation between daily activities, depression, and anxiety (45–48). A nationwide survey in South Korea found that individuals with severely restricted daily activities had more than twice the risk of depression than those with unchanged or slightly restricted activities (46). Studies before the COVID-19 pandemic demonstrated that restricted daily activities predicted suicidal ideation in women (49), whereas participation in productive activities reduced suicidal ideation in older women (50).

Additional analyses were conducted to explore the impact of specific activities on mental health. Sleep and physical and social activities exhibited a good model fit and significant reciprocal paths toward depressive symptoms. Poor sleep quality was correlated with greater depressive symptoms and continued poor sleep quality in subsequent assessments. Similarly, depression affected sleep quality and led to further depressive symptoms. This finding aligns with the findings of prior studies. To be specific, participants exhibiting poor sleep quality reported elevated levels of anxiety and depression (51, 52). Similarly, lower physical activity predicted depressive symptoms, and greater depressive symptoms predicted lower physical activity. Social activities and depression exhibited negative cross-lagged effects, with reduced social activities predicting subsequent depression, and vice versa. This aligns with previous research demonstrating the influence of social network size on depressive symptoms over four waves during COVID-19, where smaller social networks were linked to greater loneliness, which in turn predicted higher depression and further reductions in social network size (53).

The CLPM results for anxiety and daily activities exhibited a good fit. Higher levels of anxiety were associated with poorer sleep and diet quality and decreased physical activity. However, sleep quality, diet, and physical activity did not significantly impact the severity of anxiety. Similarly, previous studies have identified a significant negative correlation between sleep quality and anxiety (54). Social activities and anxiety exhibited significant autoregressive and cross-lagged effects over the seven-month study period. Low social activities contributed to higher anxiety, and higher anxiety predicted fewer social activities. Similarly, a previous study demonstrated that low social activities were linked to greater anxiety during the COVID-19 quarantine (55).

In the suicidality model, satisfactory fit indices to identify the reciprocal effects between daily activities and suicidality were not achieved. Only social activities exhibited significant cross-lagged paths toward suicidality. Previous studies have shown that decreased social networks and relationships, along with loneliness and living in solitude, are strongly associated with suicidal ideation and behavior (30, 56). Prolonged social distancing exacerbates these risks, with loneliness being a long-term risk factor (57).

We explored the differences in daily activities and found that women were more likely to engage in social activities than men. In an analysis of gender differences across 59 countries, a previous study found that women were more inclined to seek emotional support as a coping mechanism compared to men during the COVID-19 pandemic (58). The current study also found that lower income and educational status were correlated with higher depression symptoms, whereas only income was correlated with anxiety. None of the demographic variables were significantly correlated with suicidality in our models. Previous studies have linked socioeconomic status, including education, income, and occupational skills, to major depressive disorder (59).

Our findings confirm the reciprocal relationship between daily activities and mental health, where disruptions in daily activities—often indicated as signs of functional impairment in diagnostic criteria for mental disorders—can either worsen or mitigate mental health issues. Behavioral Activation (BA), an intervention that emphasizes monitoring and increasing engagement in important activities, has been shown to enhance this relationship (60). According to a meta-analysis, BA outperformed inactive controls in improving depression with a large effect size, anxiety with a small effect size, and increasing activation with a moderate effect size (61).

The reciprocal relationship between daily activities and mental health has been explored before the COVID-19 era (62–64). The present study corroborates the relationship between daily activities and mental health persisted even through the pandemic’s changing circumstances. The findings of previous studies examining the association between daily activities and mental health are consistent with our results, supporting the robustness of this relationship across different contexts. However, the effects of restrictions on daily activities during the pandemic and their potential impacts in the post-pandemic period require further investigation.

This study had several advantages over previous studies. First, it used a large, stratified sample and achieved greater representativeness. While many prior studies predominantly focused on older adults, this study encompassed participants from a wide range of age groups. Furthermore, our research provides a more detailed understanding of how daily activities impact mental states across diverse populations by controlling demographic variables such as age, gender, income, and educational status. This methodological rigor goes beyond cross-sectional studies to further elucidate complex reciprocal relationships between daily activities and mental health (65). Examining both the overall activities and each specific activity, these results are expected to stimulate future research about the relationship between activities and mental health.

Beyond analyzing overall daily activities, identifying populations with reduced activity levels at an early stage and the following changes in mental health emphasize the importance of the development and application of early interventions. This study explored the impact of specific activities on mental health, providing valuable insights into which activities are beneficial for various demographic groups. This information can be used to provide tailored behavioral strategies in clinical settings.

However, this study had several limitations. First, it relied on self-reported data on daily activities and mental states. In future research, various methods such as ecological momentary assessments can be used. Second, we investigated the relationship between daily activities and mental health for 7 months. Future research should explore the sustained negative impacts in the post-pandemic era. Finally, our results should be replicated with a larger sample size. Although physical and learning activities did not show significant paths in this study, previous research has indicated their impact on mental health during the pandemic (66).

## Conclusion

Maintaining a balanced level of core daily activities is crucial for maintaining mental health. Tailored behavioral activation interventions for individual types of activities may be beneficial. Activities should be promoted equitably by considering differences in core activities based on demographic factors.

## Data Availability

The datasets presented in this article are not readily available because the data set belongs to the Korea University SMI laboratory and is accessible to relevant researchers. Requests to access the datasets should be directed to Juhee Choi, cjhee0112@gmail.com.
