# Supplementary material for: Longitudinal relationships between daily activities, depressive symptoms, anxiety, and suicidality during the COVID-19 pandemic: a three-wave cross-lagged study
Source: Front Public Health. 2025 Mar 11;13:1459300. doi: 10.3389/fpubh.2025.1459300 (PMC11932851; doi:10.3389/fpubh.2025.1459300)
Supplement: Supplementary file 1 [file Table_1.DOCX]

Supplementary Material

Table S1. Mental health measures cut-off and prevalence

|  |  |  | **Total sample (N=586)** | | |
| --- | --- | --- | --- | --- | --- |
| **Measure** | **Cut-off points** | **Meaning** | **Prevalence N (%)** | | |
|  |  |  | T1 | T2 | T3 |
| **MHS: D** | 0~8 | No symptoms | 363(61.95%) | 358(61.09%) | 385(65.7%) |
|  | 9~12 | Mild | 60(10.24%) | 85(14.51%) | 64(10.92%) |
|  | 13~16 | Moderate | 66(11.26%) | 65(11.09%) | 71(12.12%) |
|  | 17~20 |  |  |  |  |
|  | ≥21 | Severe | 97(16.55%) | 78(13.31%) | 66(11.26%) |
|  |  |  |  |  |  |
| **MHS: A** | 0~9 | No symptoms | 358(61.09%) | 384(65.53%) | 380(64.85%) |
|  | 10~14 | Mild | 74(12.63%) | 60(10.24%) | 79(13.48%) |
|  | 15~19 |  |  |  |  |
|  | 20~29 | Moderate | 126(21.5%) | 113(19.28%) | 106(18.09%) |
|  | ≥30 | Severe | 28(4.78%) | 29(4.95%) | 21(3.58%) |
|  |  |  |  |  |  |
| **MHS: S** | 0 | No symptoms | 425(72.53%) | 430(73.38%) | 429(73.21%) |
|  | 1 | Mild | 49(8.36%) | 61(10.41%) | 46(7.85%) |
|  | 2 | Moderate | 25(4.27%) | 17(2.9%) | 36(6.14%) |
|  | ≥3 | Severe | 87(14.85%) | 78(13.31%) | 75(12.8%) |

Table S2. Model Fit Indices for alignment measurement invariance

| **Model specification** | ***df*** | ***χ2*** | ***p*** | **CFI** | **SRMR** | **RMSEA** |
| --- | --- | --- | --- | --- | --- | --- |
| Depressive symptoms |  |  |  |  |  |  |
| Configural invariance | 162 | 1879.107 | <.001 | 0.901 | 0.043 | 0.134 |
| Metric invariance | 184 | 1892.667 | <.001 | 0.901 | 0.045 | 0.126 |
| Model difference | 22 | 13.560 | .916 |  |  |  |
| Anxiety symptoms |  |  |  |  |  |  |
| Configural invariance | 132 | 1528.371 | <.001 | 0.932 | 0.033 | 0.134 |
| Metric invariance | 152 | 1543.698 | <.001 | 0.933 | 0.035 | 0.125 |
| Model difference | 20 | 15.327 | .757 |  |  |  |
| Suicidality |  |  |  |  |  |  |
| Configural invariance | 6 | 82.317 | <.001 | 0.987 | 0.015 | 0.147 |
| Metric invariance | 12 | 108.295 | <.001 | 0.984 | 0.033 | 0.117 |
| Model difference | 6 | 25.978 | <.001 |  |  |  |
| Daily activities level |  |  |  |  |  |  |
| Configural invariance | 15 | 170.859 | <.001 | 0.928 | 0.043 | 0.144 |
| Metric invariance | 23 | 173.598 | <.001 | 0.931 | 0.046 | 0.114 |
| Model difference | 8 | 2.739 | .950 |  |  |  |

Table S3. Fit indices for the CLPM Model of Mental health and specific daily activities

| **Model specification** | ***df*** | ***χ2*** | **CFI** | **SRMR** | **RMSEA** |
| --- | --- | --- | --- | --- | --- |
| **Depressive symptoms** |  |  |  |  |  |
| Sleep quality | 8 | 39.415 | 0. 978 | 0.048 | 0.082 |
| Diet quality | 8 | 41.319 | 0.979 | 0.055 | 0.084 |
| Physical Activities | 8 | 47.668 | 0.969 | 0.052 | 0.092 |
| Social Activities | 8 | 36.536 | 0.977 | 0.047 | 0.078 |
| Education | 8 | 49.920 | 0.966 | 0.053 | 0.095 |
|  |  |  |  |  |  |
| **Anxiety symptoms** |  |  |  |  |  |
| Sleep quality | 8 | 35.549 | 0.904 | 0.047 | 0.077 |
| Diet quality | 8 | 41.319 | 0. 979 | 0.055 | 0. 084 |
| Physical Activities | 8 | 43.435 | 0. 974 | 0.051 | 0.087 |
| Social Activities | 8 | 34.404 | 0.980 | 0.045 | 0.075 |
| Education | 8 | 47.143 | 0.971 | 0.052 | 0.091 |
|  |  |  |  |  |  |
| **Suicidality** |  |  |  |  |  |
| Sleep quality | 8 | 39.778 | 0.977 | 0.048 | 0.082 |
| Diet quality | 8 | 49.102 | 0.970 | 0.057 | 0.094 |
| Physical Activities | 8 | 50.865 | 0.964 | 0.052 | 0.096 |
| Social Activities | 8 | 34.861 | 0.977 | 0.048 | 0.076 |
| Education | 8 | 48.761 | 0.966 | 0.053 | 0.093 |

Table S4. Standardized Estimates for the CLPM Model of Depressive symptoms and Sleep quality, physical and social activities

| **Dependent variable** | **Independent variable** | **Estimate** | **S.E.** | **Est./S.E.** | ***p*** |
| --- | --- | --- | --- | --- | --- |
| Depression at T3 | Depression at T2 | 0.486 | 0.037 | 13.039 | <.001 |
|  | Sleep quality at T2 | -0.051 | 0.034 | -1.496 | .135 |
|  | Depression at T1 | 0.271 | 0.039 | 6.998 | <.001 |
|  | Sleep quality at T1 | -0.067 | 0.034 | -1.987 | .047 |
|  | Gender | 0.000 | 0.028 | 0.007 | .994 |
|  | Age | -0.053 | 0.028 | -1.898 | .058 |
|  | Educational status | -0.017 | 0.028 | -0.619 | .536 |
|  | Income | -0.027 | 0.028 | -0.960 | .337 |
| Sleep quality at T3 | Depression at T2 | -0.210 | 0.046 | -4.551 | <.001 |
|  | Sleep quality at T2 | 0.313 | 0.039 | 8.042 | <.001 |
|  | Depression at T1 | 0.058 | 0.046 | 1.251 | .211 |
|  | Sleep quality at T1 | 0.307 | 0.039 | 7.966 | <.001 |
|  | Gender | 0.010 | 0.033 | 0.294 | .769 |
|  | Age | 0.016 | 0.033 | 0.497 | .619 |
|  | Educational status | 0.010 | 0.033 | 0.314 | .753 |
|  | Income | 0.035 | 0.033 | 1.039 | .299 |
| Depression at T2 | Depression at T1 | 0.682 | 0.023 | 29.460 | <.001 |
|  | Sleep quality at T1 | -0.080 | 0.030 | -2.637 | .008 |
|  | Gender | 0.004 | 0.029 | 0.140 | .889 |
|  | Age | -0.008 | 0.030 | -0.271 | .786 |
|  | Educational status | -0.062 | 0.030 | -2.108 | .035 |
|  | Income | -0.043 | 0.030 | -1.437 | .151 |
| Sleep quality at T2 | Depression at T1 | -0.146 | 0.035 | -4.110 | <.001 |
|  | Sleep quality at T1 | 0.518 | 0.031 | 16.863 | <.001 |
|  | Gender | 0.027 | 0.034 | 0.795 | .427 |
|  | Age | 0.035 | 0.034 | 1.012 | .312 |
|  | Educational status | 0.007 | 0.034 | 0.219 | .827 |
|  | Income | 0.084 | 0.035 | 2.436 | .015 |
| Sleep quality at T1 WITH | Depression at T1 | -0.267 | 0.038 | -6.957 | <.001 |
| Sleep quality at T2 WITH | Depression at T2 | -0.131 | 0.041 | -3.215 | .001 |
| Sleep quality at T3 WITH | Depression at T3 | -0.117 | 0.041 | -2.871 | .004 |
| **Dependent variable** | **Independent variable** | **Estimate** | **S.E.** | **Est./S.E.** | ***p*** |
| Depression at T3 | Depression at T2 | 0.496 | 0.037 | 13.407 | <.001 |
|  | Physical activity at T2 | -0.076 | 0.031 | -2.456 | .014 |
|  | Depression at T1 | 0.289 | 0.039 | 7.486 | <.001 |
|  | Physical activity at T1 | 0.018 | 0.031 | 0.569 | .569 |
|  | Gender | -0.002 | 0.028 | -0.078 | .938 |
|  | Age | -0.04 | 0.028 | -1.406 | .160 |
|  | Educational status | -0.012 | 0.028 | -0.443 | .658 |
|  | Income | -0.038 | 0.028 | -1.333 | .183 |
| Physical activity at T3 | Depression at T2 | -0.141 | 0.049 | -2.887 | .004 |
|  | Physical activity at T2 | 0.342 | 0.037 | 9.193 | <.001 |
|  | Depression at T1 | 0.037 | 0.049 | 0.758 | .448 |
|  | Physical activity at T1 | 0.269 | 0.038 | 7.102 | <.001 |
|  | Gender | 0.048 | 0.035 | 1.372 | .170 |
|  | Age | 0.037 | 0.035 | 1.039 | .299 |
|  | Educational status | 0.008 | 0.035 | 0.217 | .828 |
|  | Income | 0.072 | 0.035 | 2.048 | .041 |
| Depression at T2 | Depression at T1 | 0.699 | 0.022 | 32.358 | <.001 |
|  | Physical activity at T1 | -0.033 | 0.03 | -1.1 | .272 |
|  | Gender | 0.006 | 0.03 | 0.212 | .832 |
|  | Age | -0.001 | 0.03 | -0.022 | .982 |
|  | Educational status | -0.062 | 0.03 | -2.082 | .037 |
|  | Income | -0.047 | 0.03 | -1.565 | .118 |
| Physical activity at T2 | Depression at T1 | -0.067 | 0.038 | -1.764 | .078 |
|  | Physical activity at T1 | 0.424 | 0.034 | 12.309 | <.001 |
|  | Gender | -0.094 | 0.038 | -2.513 | .012 |
|  | Age | 0.104 | 0.037 | 2.781 | .005 |
|  | Educational status | 0.032 | 0.037 | 0.869 | .385 |
|  | Income | 0 | 0.038 | 0.006 | .995 |
| Physical activity at T1 WITH | Depression at T1 | -0.15 | 0.04 | -3.716 | <.001 |
| Physical activity at T2 WITH | Depression at T2 | -0.104 | 0.041 | -2.533 | .011 |
| Physical activity at T3 WITH | Depression at T3 | -0.06 | 0.041 | -1.462 | .144 |
| Depression at T3 | Depression at T2 | 0.485 | 0.037 | 13.09 | <.001 |
|  | Social activity at T2 | -0.079 | 0.031 | -2.604 | .009 |
|  | Depression at T1 | 0.280 | 0.038 | 7.311 | <.001 |
|  | Social activity at T1 | -0.054 | 0.03 | -1.809 | .070 |
|  | Gender | 0.017 | 0.028 | 0.624 | .532 |
|  | Age | -0.047 | 0.028 | -1.69 | .091 |
|  | Educational status | -0.013 | 0.028 | -0.477 | .633 |
|  | Income | -0.019 | 0.028 | -0.687 | .492 |
| Social activity at T3 | Depression at T2 | -0.192 | 0.050 | -3.865 | <.001 |
|  | Social activity at T2 | 0.272 | 0.038 | 7.121 | <.001 |
|  | Depression at T1 | 0.018 | 0.050 | 0.353 | .724 |
|  | Social activity at T1 | 0.234 | 0.038 | 6.155 | <.001 |
|  | Gender | 0.079 | 0.035 | 2.228 | .026 |
|  | Age | 0.051 | 0.036 | 1.441 | .150 |
|  | Educational status | -0.003 | 0.036 | -0.078 | .938 |
|  | Income | 0.107 | 0.036 | 2.949 | .003 |
| Depression at T2 | Depression at T1 | 0.691 | 0.022 | 31.424 | <.001 |
|  | Social activity at T1 | -0.077 | 0.03 | -2.604 | .009 |
|  | Gender | 0.013 | 0.029 | 0.452 | .651 |
|  | Age | -0.004 | 0.03 | -0.132 | .895 |
|  | Educational status | -0.062 | 0.03 | -2.092 | .036 |
|  | Income | -0.04 | 0.03 | -1.334 | .182 |
| Social activity at T2 | Depression at T1 | -0.128 | 0.038 | -3.343 | .001 |
|  | Social activity at T1 | 0.365 | 0.036 | 10.232 | <.001 |
|  | Gender | 0.101 | 0.037 | 2.712 | .007 |
|  | Age | 0.037 | 0.038 | 0.986 | .324 |
|  | Educational status | 0.047 | 0.038 | 1.241 | .215 |
|  | Income | 0.118 | 0.038 | 3.094 | .002 |
| Social activity at T1 WITH | Depression at T1 | -0.169 | 0.04 | -4.202 | <.001 |
| Social activity at T2 WITH | Depression at T2 | -0.099 | 0.041 | -2.413 | .016 |
| Social activity at T3 WITH | Depression at T3 | -0.092 | 0.041 | -2.237 | .025 |

Table S5. Standardized Estimates for the CLPM Model of Anxiety symptoms and social activities

| **Dependent variable** | **Independent variable** | **Estimate** | **S.E.** | **Est./S.E.** | ***p*** |
| --- | --- | --- | --- | --- | --- |
| Anxiety at T3 | Anxiety at T2 | 0.449 | 0.038 | 11.833 | <.001 |
|  | Social activity at T2 | -0.069 | 0.029 | -2.350 | .019 |
|  | Anxiety at T1 | 0.346 | 0.039 | 8.923 | <.001 |
|  | Social activity at T1 | -0.035 | 0.029 | -1.21 | .226 |
|  | Gender | 0.016 | 0.027 | 0.594 | .553 |
|  | Age | -0.019 | 0.027 | -0.717 | .473 |
|  | Educational status | -0.023 | 0.027 | -0.873 | .382 |
|  | Income | -0.032 | 0.027 | -1.183 | .237 |
| Social activity at T3 | Anxiety at T2 | -0.151 | 0.053 | -2.866 | .004 |
|  | Social activity at T2 | 0.282 | 0.038 | 7.386 | <.001 |
|  | Anxiety at T1 | 0.006 | 0.053 | 0.119 | .905 |
|  | Social activity at T1 | 0.238 | 0.038 | 6.238 | <.001 |
|  | Gender | 0.086 | 0.036 | 2.403 | .016 |
|  | Age | 0.057 | 0.036 | 1.604 | .109 |
|  | Educational status | 0.005 | 0.036 | 0.145 | .884 |
|  | Income | 0.113 | 0.037 | 3.093 | .002 |
| Anxiety at T2 | Anxiety at T1 | 0.730 | 0.02 | 37.016 | <.001 |
|  | Social activity at T1 | -0.059 | 0.028 | -2.085 | .037 |
|  | Gender | 0.050 | 0.028 | 1.802 | .072 |
|  | Age | 0.006 | 0.028 | 0.211 | .833 |
|  | Educational status | -0.038 | 0.028 | -1.342 | .179 |
|  | Income | -0.017 | 0.028 | -0.601 | .548 |
| Social activity at T2 | Anxiety at T1 | -0.128 | 0.038 | -3.374 | .001 |
|  | Social activity at T1 | 0.366 | 0.036 | 10.258 | <.001 |
|  | Gender | 0.106 | 0.037 | 2.838 | .005 |
|  | Age | 0.041 | 0.038 | 1.083 | .279 |
|  | Educational status | 0.050 | 0.038 | 1.324 | .186 |
|  | Income | 0.120 | 0.038 | 3.136 | .002 |
| Social activity at T1 WITH | Anxiety at T1 | -0.160 | 0.040 | -3.977 | <.001 |
| Social activity at T2 WITH | Anxiety at T2 | -0.052 | 0.041 | -1.250 | .211 |
| Social activity at T3 WITH | Anxiety at T3 | -0.100 | 0.041 | -2.457 | .014 |

Table S6. Standardized Estimates for the CLPM Model of Suicidality and social activities

| **Dependent variable** | **Independent variable** | **Estimate** | **S.E.** | **Est./S.E.** | ***p*** |
| --- | --- | --- | --- | --- | --- |
| Suicidality at T3 | Suicidality at T2 | 0.449 | 0.038 | 11.954 | <.001 |
|  | Social activity at T2 | -0.075 | 0.031 | -2.445 | .014 |
|  | Suicidality at T1 | 0.341 | 0.039 | 8.793 | <.001 |
|  | Social activity at T1 | 0.015 | 0.031 | 0.503 | .615 |
|  | Gender | <.001 | 0.028 | 0.016 | .987 |
|  | Age | -0.022 | 0.028 | -0.779 | .436 |
|  | Educational status | 0.007 | 0.028 | 0.239 | .811 |
|  | Income | 0.003 | 0.029 | 0.102 | .919 |
| Social activity at T3 | Suicidality at T2 | -0.142 | 0.05 | -2.846 | .004 |
|  | Social activity at T2 | 0.299 | 0.038 | 7.868 | <.001 |
|  | Suicidality at T1 | 0.018 | 0.051 | 0.35 | .727 |
|  | Social activity at T1 | 0.243 | 0.038 | 6.365 | <.001 |
|  | Gender | 0.063 | 0.036 | 1.76 | .078 |
|  | Age | 0.061 | 0.036 | 1.695 | .090 |
|  | Educational status | -0.001 | 0.036 | -0.028 | .978 |
|  | Income | 0.114 | 0.037 | 3.093 | .002 |
| Suicidality at T2 | Suicidality at T1 | 0.698 | 0.021 | 32.553 | <.001 |
|  | Social activity at T1 | -0.042 | 0.03 | -1.413 | .158 |
|  | Gender | 0.030 | 0.029 | 1.026 | .305 |
|  | Age | -0.002 | 0.03 | -0.070 | .945 |
|  | Educational status | -0.029 | 0.03 | -0.982 | .326 |
|  | Income | 0.022 | 0.03 | 0.737 | .461 |
| Social activity at T2 | Suicidality at T1 | -0.107 | 0.038 | -2.818 | .005 |
|  | Social activity at T1 | 0.376 | 0.035 | 10.648 | <.001 |
|  | Gender | 0.083 | 0.038 | 2.205 | .027 |
|  | Age | 0.047 | 0.038 | 1.238 | .216 |
|  | Educational status | 0.041 | 0.038 | 1.085 | .278 |
|  | Income | 0.116 | 0.039 | 3.001 | .003 |
| Social activity at T1 WITH | Suicidality at T1 | -0.114 | 0.041 | -2.799 | .005 |
| Social activity at T2 WITH | Suicidality at T2 | 0.056 | 0.041 | 1.351 | .177 |
| Social activity at T3 WITH | Suicidality at T3 | -0.034 | 0.041 | -0.823 | .410 |
